# Cerebrovascular lesion loads and accelerated brain aging: insights into the cognitive spectrum

**DOI:** 10.3389/frdem.2024.1380015

**Published:** 2024-06-21

**Authors:** Iman Beheshti, Olivier Potvin, Mahsa Dadar, Simon Duchesne

**Affiliations:** ^1^Department of Human Anatomy and Cell Science, Rady Faculty of Health Sciences, University of Manitoba, Winnipeg, MB, Canada; ^2^Centre de recherche CERVO, Québec, QC, Canada; ^3^Centre de recherche de l'Institut universitaire de cardiologie et pneumologie de Québec, Québec, QC, Canada; ^4^Department of Psychiatry, McGill University, Montreal, QC, Canada; ^5^Douglas Mental Health University Institute, McGill University, Montreal, QC, Canada; ^6^Département de Radiologie et de Médecine Nucléaire, Faculté de Médecine, Université Laval, Québec, QC, Canada

**Keywords:** brain age estimation, anatomical MRI, Alzheimer's disease, cerebral microbleeds, vascular dementia, white matter hyperintensities, machine learning

## Abstract

**Introduction:**

White matter hyperintensities (WMHs) and cerebral microbleeds are widespread among aging population and linked with cognitive deficits in mild cognitive impairment (MCI), vascular MCI (V-MCI), and Alzheimer's disease without (AD) or with a vascular component (V-AD). In this study, we aimed to investigate the association between brain age, which reflects global brain health, and cerebrovascular lesion load in the context of pathological aging in diverse forms of clinically-defined neurodegenerative conditions.

**Methods:**

We computed brain-predicted age difference (brain-PAD: predicted brain age minus chronological age) in the Comprehensive Assessment of Neurodegeneration and Dementia cohort of the Canadian Consortium on Neurodegeneration in Aging including 70 cognitively intact elderly (CIE), 173 MCI, 88 V-MCI, 50 AD, and 47 V-AD using T1-weighted magnetic resonance imaging (MRI) scans. We used a well-established automated methodology that leveraged fluid attenuated inversion recovery MRIs for precise quantification of WMH burden. Additionally, cerebral microbleeds were detected utilizing a validated segmentation tool based on the ResNet50 network, utilizing routine T1-weighted, T2-weighted, and T2^*^ MRI scans.

**Results:**

The mean brain-PAD in the CIE cohort was around zero, whereas the four categories showed a significantly higher mean brain-PAD compared to CIE, except MCI group. A notable association trend between brain-PAD and WMH loads was observed in aging and across the spectrum of cognitive impairment due to AD, but not between brain-PAD and microbleed loads.

**Discussion:**

WMHs were associated with faster brain aging and should be considered as a risk factor which imperils brain health in aging and exacerbate brain abnormalities in the context of neurodegeneration of presumed AD origin. Our findings underscore the significance of novel research endeavors aimed at elucidating the etiology, prevention, and treatment of WMH in the area of brain aging.

## Highlights

- We assessed the association between brain-PAD and cerebrovascular lesion loads in aging and AD.
- There were noticeably links between brain-PAD and WMH loads.
- The structure of the aging brain is associated with WMHs.
- WMH needs to be taken into account as a risk factor that increases brain age in aging and AD.

## 1 Introduction

Assessing brain health status using machine learning models is a topic of increasing interest, with a number of useful applications being proposed such as monitoring brain aging and quantifying the impact of neurodegeneration (Mishra et al., 2021). A recent addition consists in predicting a “brain age” metric as an indicator of cerebral health (Franke and Gaser, 2019; Mishra et al., 2021; Sone and Beheshti, 2022), whereby observable characteristics from neuroimaging—e.g., cortical thickness as measured on magnetic resonance imaging (MRI)—are used as dependent variables in an estimation framework of a group of individual's chronological age (Pardoe and Kuzniecky, 2018). This allows the generation of a brain age estimate for any new individual based on similar characteristics, with any discrepancy between chronological and predicted brain ages being seen as a departure from the norm defined by the initial training set.

In the area of Alzheimer's disease (AD), the brain age paradigm has been used to uncover its association with traditional neuropsychological screening tools (Beheshti et al., 2018), predict the conversion of mild cognitive impairment (MCI) to probable AD (Gaser et al., 2013), and study the trajectory of metabolism along the cognitive impairment spectrum (Beheshti et al., 2021). In fact, cortical thickness-based brain age has been shown to be a stronger predictor of cognitive impairment than chronological age (Habes et al., 2021). However, these previous studies have only focused on the spectrum of dementia from probable AD. Clinical-pathological studies have shown however that, while being the most frequent pathology, the incidence of “pure” AD is low (around 6% of individuals with a major neurocognitive disorder) (Boyle et al., 2017a). In fact, more than three-quarters of individuals exhibit two or more pathologies at death, the most prevalent combination being AD and vascular pathology (Boyle et al., 2017b). Rather than the exception, mixed presentations are therefore the norm and should be studied together whenever possible.

The clinical diagnostic of vascular MCI (V-MCI) and mixed vascular-AD dementia (V-AD) relies on a series of clinical criteria (see Section 2.2.2) as well as on the presence of cerebrovascular lesions on computer tomography or MRI scans, such as microbleeds and white matter hyperintensities (WMH). The latter appear on T2-weighted and fluid attenuated inversion recovery (FLAIR) scans and are typically seen in the aging population (Dadar et al., 2021a). The prevalence of WMH is 10–20% for people in their 60 s, reaching 100% in people over the age of 90 (Smith et al., 2017). In the aging population, WMHs can contribute to a higher rate of brain atrophy in beyond-normal brain aging, particularly in regions related to AD (Habes et al., 2016), such as the medial temporal lobe, insular lobe, and temporal lobe (Cao et al., 2022).

WMHs are often caused by small vessel disease, with some being associated with small subcortical infarcts. The manifestation of WMHs has been associated with a broad spectrum of histological alterations, including demyelination and axonal loss, diminished glial density and atrophy, endothelial and immune activation, ischemic damage, hypoxia and hypoperfusion, and, critical to this work, cortical thinning and cerebral atrophy (Seo et al., 2012a,b). WMH are reported to arise from incomplete infarction, indicating a prolonged reduction in blood flow in deep brain regions due to conditions such as arteriolosclerosis, lipohyalinosis, and fibrinoid necrosis affecting small brain arterioles and arteries (Merino, 2019). This reduced blood flow would lead to changes in oxygen levels, disrupts the brain's ability to regulate blood flow, and triggering the activation of genes that promote inflammation. As a result, the blood-brain barrier would become compromised, allowing inflammatory proteins to enter vessel walls and brain tissue. These series of events ultimately would lead to the breakdown of myelin, damage to axons, reduced glial cell density, vacuolation, and cortical atrophy (Rosenberg et al., 2016; Merino, 2019).

Recent studies have revealed a pervasive presence of WMH among patients with diverse neurodegenerative disorders, encompassing conditions such as MCI and AD; those with an associated vascular component, V-MCI and V-AD; cognitively intact elderly with Parkinson's disease; cognitively impaired Parkinson's disease; frontotemporal dementia; Lewy body dementia; and mixed dementias (Dadar et al., 2021a). Remarkably, In the context of AD, it has been shown that a heavy burden of WMH can lead to an elevated risk of dementia due to AD and a faster progression from intact cognition to MCI (Soldan et al., 2020). Likewise, a similar narrative unfolds regarding the burden of microbleeds, wherein their presence can have a detrimental effect on cognitive functioning and can make individuals more vulnerable to dementia (Greenberg et al., 2009; Puy et al., 2021).

In this study, we aimed to explore brain aging in various populations along both spectra of cognition and cerebrovascular disease, as evidenced by the presence of a cerebrovascular lesion load that includes both WMHs and microbleeds. We also analyzed individuals with varying levels of cerebrovascular lesion load to determine the impact of these lesions on overall brain health.

In view of the relevant literature, we hypothesized that (1) vascular cohorts (i.e., V-MCI and V-AD) would experience a notably accelerated brain aging process compared to non-vascular cohorts (i.e., MCI and AD), and (2) the presence of cerebrovascular lesions would be associated with an elevated brain age not only in individuals with AD, but also in cognitively healthy older adults.

To this end, we estimated cortical morphometric-based brain age in a large group of participants from the Comprehensive Assessment of Neurodegeneration and Dementia (COMPASS-ND) study that included cognitively intact elderly (CIE), individuals with MCI and probable AD, but also participants with V-MCI and V-AD, with specific attention to the associations between brain age and cerebrovascular lesions.

## 2 Material and methods

### 2.1 Ethical agreement

Ethical agreements were obtained at all respective sites. Written informed consent was obtained from all participants.

### 2.2 Participants

#### 2.2.1 Training set

The data used to train the brain age estimate model were obtained from CIE participants enrolled in the Open Access Series of Imaging Studies (OASIS), Alzheimer's Disease Neuroimaging Initiative (ADNI), Banner Alzheimer's Institute (BAI), and Alzheimer's Disease Repository Without Borders (ARWIBO) studies. All CIE participants used in the training set were free from any indications of cognitive impairment or neurological disorders as per the criteria outlined in the databases.

ADNI (adni.loni.usc.edu) was launched in 2003 as a public-private partnership led by Principal Investigator Michael W. Weiner, MD. The primary goal of ADNI has been to test whether serial MRI, positron emission tomography, other biological markers, and clinical and neuropsychological assessment can be combined to measure the progression of MCI and early AD. ADNI was carried out with the goal of recruiting 800 adults aged from 55 to 90 years and consists of approximately 200 cognitively normal patients, 400 patients with MCI, and 200 patients with AD.

#### 2.2.2 Test set

The data on our test set participants were collected in the Canada-wide multi-center, prospective, longitudinal COMPASS-ND cohort study of the Canadian Consortium for Neurodegeneration and Aging (CCNA; https://ccna-ccnv.ca/compass-nd-study/) (Chertkow et al., 2019), a national initiative to catalyze research on dementia. The overall study design and methods have been published previously (Chertkow et al., 2019). The study is registered on clinicaltrials.gov (NCT03402919). COMPASS-ND includes deeply phenotyped participants with various forms of dementia and mild memory loss or concerns, along with CIE. Clinical diagnoses were determined by participating clinicians based on longitudinal clinical, screening, and MRI findings (i.e., diagnosis reappraisal was performed using information from recruitment assessment, screening visit, clinical visit with physician input, and MRI). In particular, criteria for V-MCI were derived from consensus criteria from the American Heart Association (Gorelick et al., 2011) and International Society for Vascular Cognitive and Behavioral Disorders (Sachdev et al., 2014). V-MCI participants were required to be age ≥60, have MCI according to National Institute on Aging-Alzheimer's Association criteria (Albert et al., 2011), not have a prior history of clinical stroke, and to have evidence of cerebrovascular disease on brain MRI defined as two or more supratentorial infarcts (i.e., excluding brainstem or cerebellar infarcts) or beginning confluent or confluent WMH. Criteria for mixed dementia were adapted from National Institute on Aging-Alzheimer's Association criteria for dementia due to AD (McKhann et al., 2011) and required that a non-AD cause of dementia should additionally be present.

This cohort included COMPASS-ND participants for whom T1-weighted, T2-weighted, T2^*^, and FLAIR MRI were obtained. Of note, these data were completely independent from the data used for training the brain age algorithm.

### 2.3 Image acquisition and processing

The acquisition of COMPASS-ND MRIs was done according to the Canadian Dementia Imaging Protocol (CDIP; https://www.cdip-pcid.ca) (Duchesne et al., 2019). T1-weighted images were used to extract cortical thickness measurements from which brain age was derived. To this end, we utilized the *FreeSurfer* version 6.0 segmentation software (http://surfer.nmr.mgh.harvard.edu) and the Desikan-Killiany-Touville atlas (Klein and Tourville, 2012) to extract neocortical measurements (i.e., surface, volume and thickness extracted from *aparc.DKTatlas* files). Each brain segmentation was visually inspected through at least 20 evenly distributed coronal sections. This procedure was applied to both training and test sets.

WMH load was extracted from T2w-FLAIR images from the COMPASS-ND study. We used a previously validated technique which segments WMHs in native FLAIR space and generates total WMH loads (Dadar et al., 2017), publicly available at: http://nist.mni.mcgill.ca/white-matter-hyperintensities/. For each participant, WMH load was quantified as the volume of voxels that have been categorized as WMH in the standard space, adjusted for head size. The quality of WMH processing and segmentation was visually assessed for quality by one expert (M.D.), resulting in the exclusion of nine cases from the total of 976. A logarithmic transformation was implemented on WMH volumes to normalize their distribution.

The identification of cerebral microbleeds was accomplished using a validated segmentation tool that works based on the ResNet50 network and routine T1-weighted, T2-weighted, and T2^*^ MRI scans, as described in (Dadar et al., 2022b). Visual assessment of the quality of T1, T2, and T2^*^ MRI scans was conducted by the CCNA neuroimaging team prior to applying our cerebral microbleed segmentation tool. In every participant, the total number of cerebral microbleeds was extracted and then log-transformed to normalize the overall distribution. Due to their missing T2^*^-weighted MRI, twenty-two participants were excluded from cerebral microbleed analysis: five with CIE, seven with MCI, five with AD, four with V-MCI, and one with V-AD.

### 2.4 Brain age estimation model

A standard linear support vector regression algorithm conducted in MATLAB (i.e., “fitrsvm” function, kernel: linear) was used to estimate brain age. Chronological age was considered the dependent variable, and anatomical measurements extracted from *FreeSurfer* segmentation along with sex and total intracranial volume were the independent variables, in total *n* = 188 features per individual. Our brain age prediction model was based on cortical mean thickness, volume, and surface measurements, omitting any white matter related features. We used the ComBat technique implemented in MATLAB to harmonize data from different scanners (Fortin et al., 2018). First, we assessed the accuracy of the prediction model on the training data set (*N* = 1,627, mean age = 67.75 ± 9.53 years, 56% females) through a 10-fold cross-validation strategy. The prediction accuracy was measured on the basis of the coefficient of determination (*R*^2^) between chronological and estimated age, the mean absolute error (MAE), and root mean square error (RMSE). The brain-PAD (i.e., predicted brain age minus real age) was also calculated. Bias adjustment was applied to remove the age-dependency on the predicted values (Beheshti et al., 2019) (https://github.com/medicslab/Bias_Correction). Next, the final prediction model was developed with the entire training set (*N* = 1,627). Of note, a positive brain-PAD (strictly speaking, cortical brain-PAD) stands for older-appearing cortices (i.e., estimated age > chronological age), whereas a negative brain-PAD value stands for younger-appearing cortices (i.e., estimated age <chronological age).

### 2.5 Statistical analysis

The brain age prediction model was applied to COMPASS-ND participants. As the test groups had different age and sex distributions, we regressed out the effects of age and sex from brain-PAD, WMH, and cerebral microbleed values by referencing the CIE group (*N* = 70) as follows:


(1) $$\begin{array}{l} Variable_{adjusted\;subject}=Variable_{raw\;subject}-\beta_{1}(Age_{subject}-\\ \bar{Age_{CIE}})-\beta_{2}(Sex_{subject}-\bar{Sex_{CIE}}) \end{array}$$

Where β_1_ and β_2_ stand for the slopes of the linear regression lines between age and the variable of interest, and between sex and the variable of interest, respectively, in the CIE group. Additionally, $\bar{Age_{CIE}}$ and $\bar{Sex_{CIE}}$ represent the mean age and mean sex for all CIEs, respectively.

The mean adjusted brain-PAD and WMHs for each group (CIE, MCI, AD, V-MCI, V-AD) were examined using an analysis of covariance (ANOVA). The *p*-values were adjusted using Bonferroni correction. Pearson correlation tests were utilized to assess the associations between adjusted brain-PAD and adjusted WMH, as well as between adjusted brain-PAD and adjusted microbleed loads. For all statistical tests, *P* < 0.05 was considered as significant.

## 3 Results

### 3.1 Demographics

The training set was composed of *n* = 1,627 CIE (mean age ± sd: 67.7 ± 9.5, age range: 50–94, 915 females). The test set was composed of 70 CIE, 173 MCI, 50 V-MCI, 88 AD, and 47 V-AD participants. Table 1 shows demographics for our different groups. Of note, mean age was different across diagnostic groups.

**Table 1:** Clinical demographics, WMH load, Microbleed count, and brain-PAD by diagnosis.

	**CIE (*N* = 70)**	**MCI (*N* = 173)**	**AD (*N* = 50)**	**V-MCI (*N* = 88)**	**V-AD (*N* = 47)**	***P***
Female (%)	78%	45%	36%	34%	49%	<0.0001
Real age (yrs)	69.8 ± 6.6	71.8 ± 6.6	73.9 ± 8.2	76.1 ± 5.5	76.7 ± 6.3	<0.0001
MoCa	27.7 ± 1.55	23.61 ± 3.12	18.68 ± 3.72	23.15 ± 3.22	17.78 ± 3.30	<0.0001
WMH load^†^	0.61 ± 0.5^a^	0.63 ± 0.36^c^	0.79 ± 0.37	1.97 ± 1.16^f^	2.16 ± 1.38^f^	<0.0001
WMH load^††^	0.61 ± 0.4^a^	0.55 ± 0.33^c^	0.62 ± 0.38	1.73 ± 1.09^f^	1.89 ± 1.37^f^	<0.0001
Microbleed count^†^	21.52 ± 14.24^b^	23.78 ± 14.06^d^	22.06 ± 13.14^e^	27.12 ± 18.96^f^	32.67 ± 31.50^e^	0.01
Microbleed count^††^	21.52 ± 13.98^b^	22.03 ± 14.53^d^	20.33 ± 13.52^e^	25.98 ± 18.55^f^	31.90 ± 30.55^e^	0.005
Brain-PAD (yrs)^†^	−0.52 ± 4.75	1.89 ± 5.5	7.22 ± 7.33	3.97 ± 5.43	7.64 ± 5.95	<0.0001
Brain-PAD (yrs)^††^	−0.52 ± 4.44	0.70 ± 6.03	5.86 ± 8.02	2.95 ± 5.81	7.02 ± 6.06	<0.0001

CIE, cognitively intact elderly; AD, Alzheimer's disease; MoCA, Montreal Cognitive Assessment; MCI, mild cognitive impairment; V-AD, vascular Alzheimer's disease. All variables are presented based on the mean ± standard deviation. *P*, the results of an ANOVA for continuous variables and a Chi-square test for categorical variables between groups.

† Raw data.

†† Sex- and age-adjusted data by referencing to the CIE group.

a Data missing in two participants.

b Data missing in five participants.

c Data missing in thirteen participants.

d Data missing in seven participants.

e Data missing in four participants.

f Data missing in three participants.

### 3.2 Brain age estimation performance on training set

**Figure 1:**
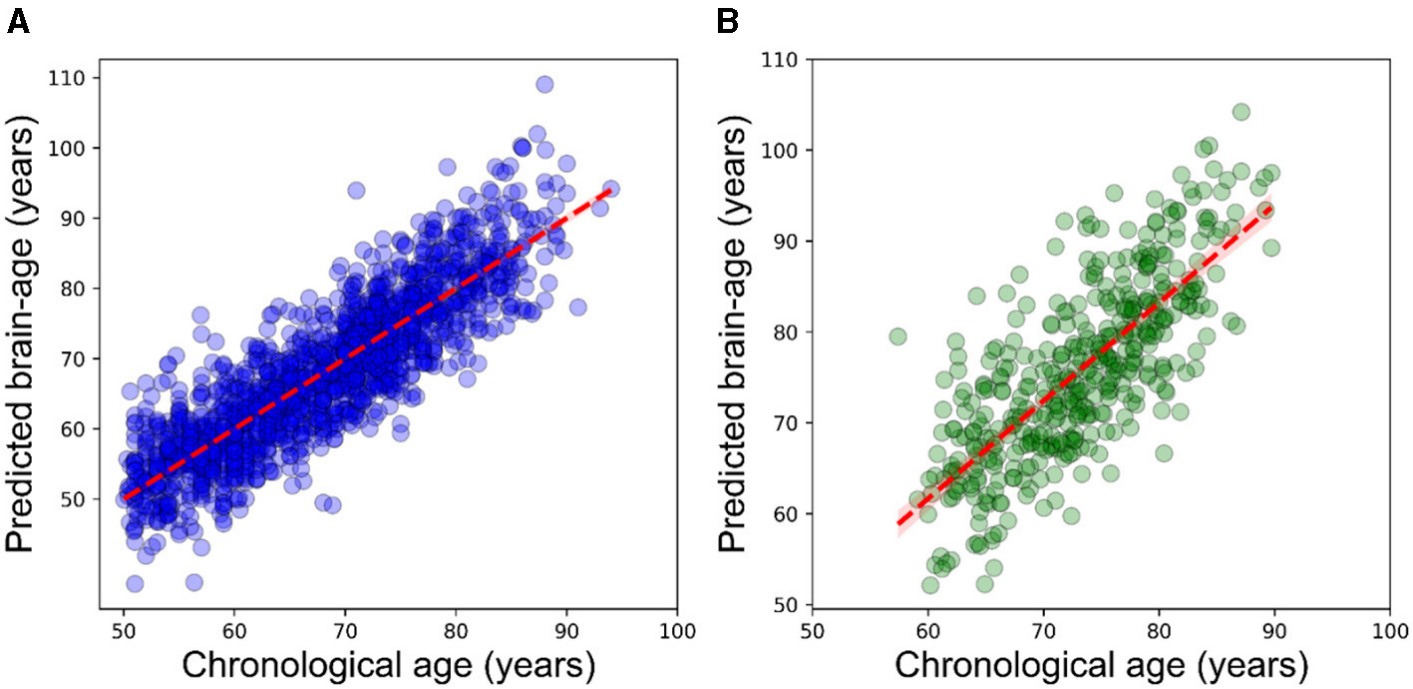
Scatterplot displaying the relationship between chronological age (*x*-axis) and estimated brain age (*y*-axis). **(A)** within the training dataset (*N* = 1,627) using the outcomes of 10-fold cross-validation, **(B)** within the CIE group. The dashed line (red) represents the regression line.

Our prediction model showed a desirable performance on the training set (*N* = 1,627) followed by 10-fold cross-validation (*R*^2^ = 0.77, MAE = 4.2 years, RMSE = 5.4 years, mean brain-PAD = 0 ± 4.8 years, Figure 1A).

### 3.3 Brain age estimation on the test set

**Figure 2:**
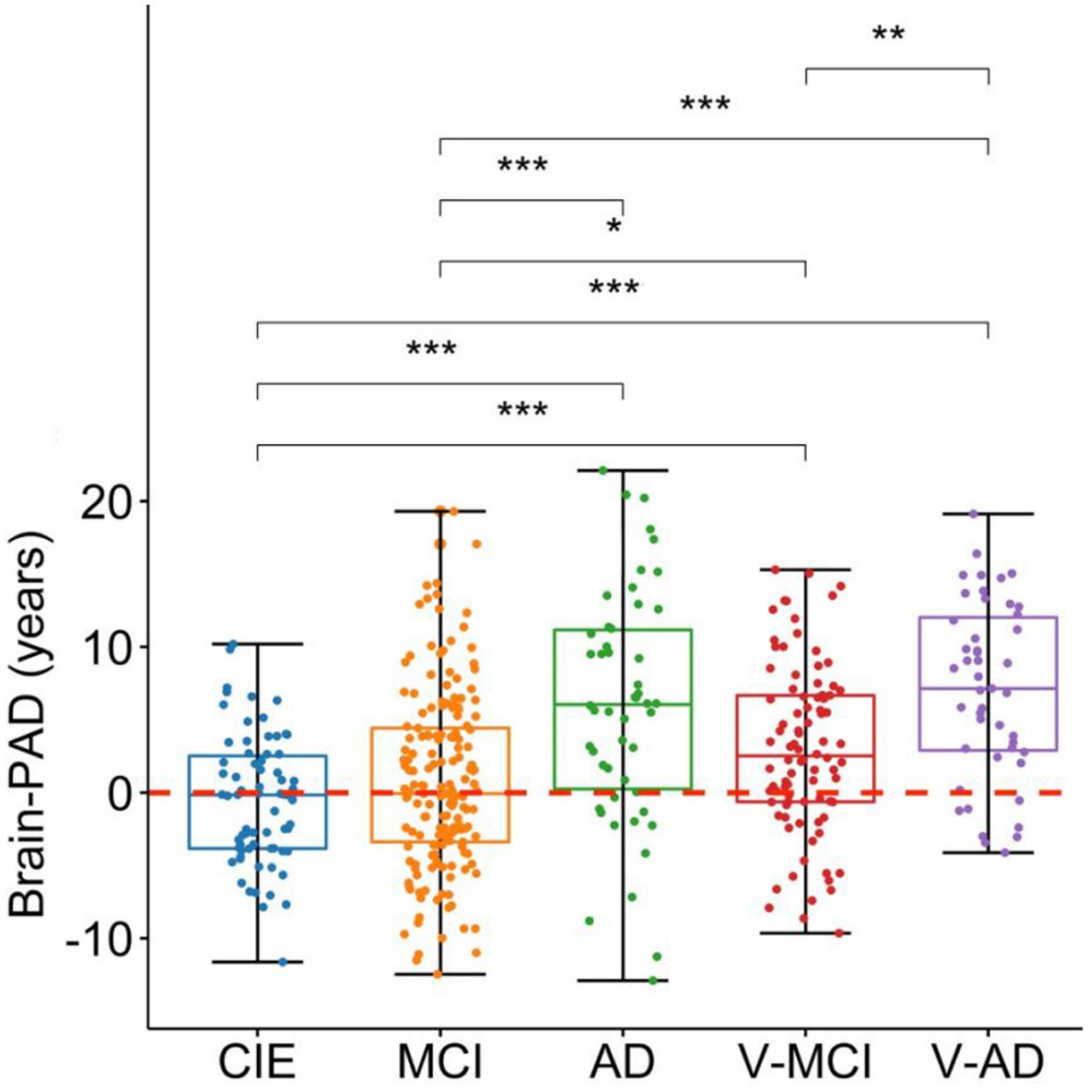
Boxplots depicting the adjusted brain-PAD values among different cohorts. CIE, cognitively intact elderly; MCI, mild cognitive impairment; AD, Alzheimer's dementia; V-MCI, vascular MCI (V-MCI); (AD), V-AD, vascular AD. Pairwise comparisons were conducted through ANOVA test with the *p*-value adjusted using Bonferroni correction. *P < 0.05, **P < 0.001, ***P < 0.0001. The adjusted brain-PAD values were obtained by regressing out the effects of age and sex from the raw brain-PAD values, referencing the CIE group.

The prediction performance on the CIE group was: *R*^2^ = 0.55, MAE = 5.2 years, RMSE = 6.5 years (Figure 1B). As per the initial aim of this study, we computed brain-PAD among five categories of participants. The mean brain-PAD values are shown in Table 1 and Figure 2. There was a significant difference in adjusted brain-PAD values [*F*_(4,423)_ = 18, *P* < 0.001, ANOVA test] among groups. All categories of patients exhibited a significantly higher mean adjusted brain-PAD than the CIE group (*P* < 0.001), except for the MCI cohort (*P* = 0.68). The V-AD cohort had the highest adjusted brain-PAD. *Post-hoc* pairwise group comparison based on the ANOVA test showed statistically significant differences (*P* < 0.05) in terms of adjusted brain-PAD between pair groups, except for CIE vs. MCI and V-AD vs. AD (*P* > 0.05).

### 3.4 WMH loads

**Figure 3:**
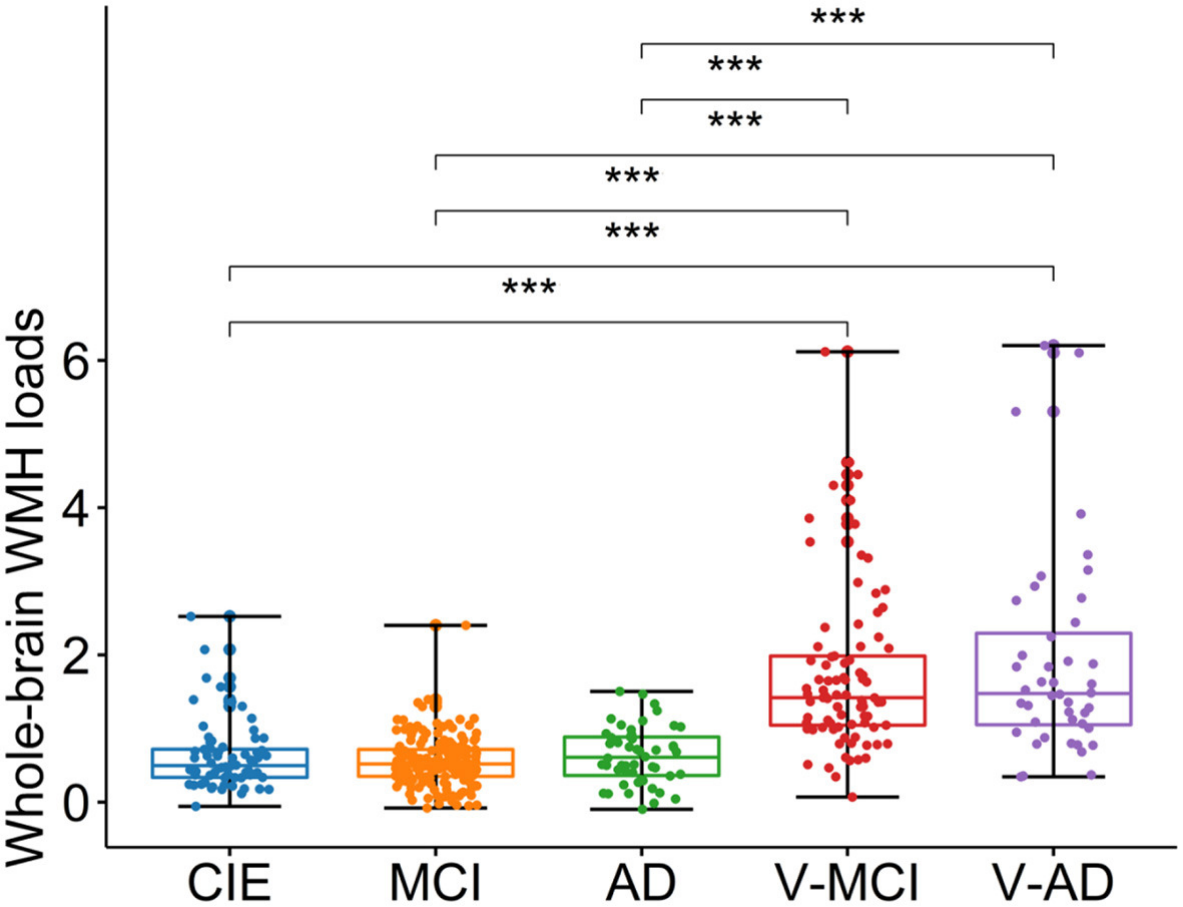
Boxplots depicting the adjusted WMH loads among different cohorts. CIE, cognitively intact elderly; MCI, mild cognitive impairment; AD, Alzheimer's dementia; V-MCI, vascular MCI (V-MCI); (AD), V-AD, vascular AD. Pairwise comparisons were conducted through ANOVA test with the *p*-value adjusted using Bonferroni correction. *P < 0.05, **P < 0.001, ***P < 0.0001. The adjusted WMH loads were obtained by regressing out the effects of age and sex from the raw WMH loads, referencing the CIE group.

Table 1 summarizes adjusted WMH loads by diagnostic category, whereas Figure 3 shows respective boxplots as well as pairwise comparisons. As could be expected, there was a significant difference in WMH loads [*F*_(4,402)_ = 58, *P* < 0.001, ANOVA test] between groups. Unsurprisingly, both V-MCI and V-AD showed a significantly higher WMH load compared to non-vascular groups (i.e., CIE, MCI, and AD) in terms of adjusted WMH loads by ANOVA pairwise comparison (*P* < 0.001). However, there were no pairwise differences between CIE vs. MCI, CIE vs. AD, MCI vs. AD, and V-MCI vs. V-AD (*P* > 0.05).

### 3.5 Microbleed counts

**Figure 4:**
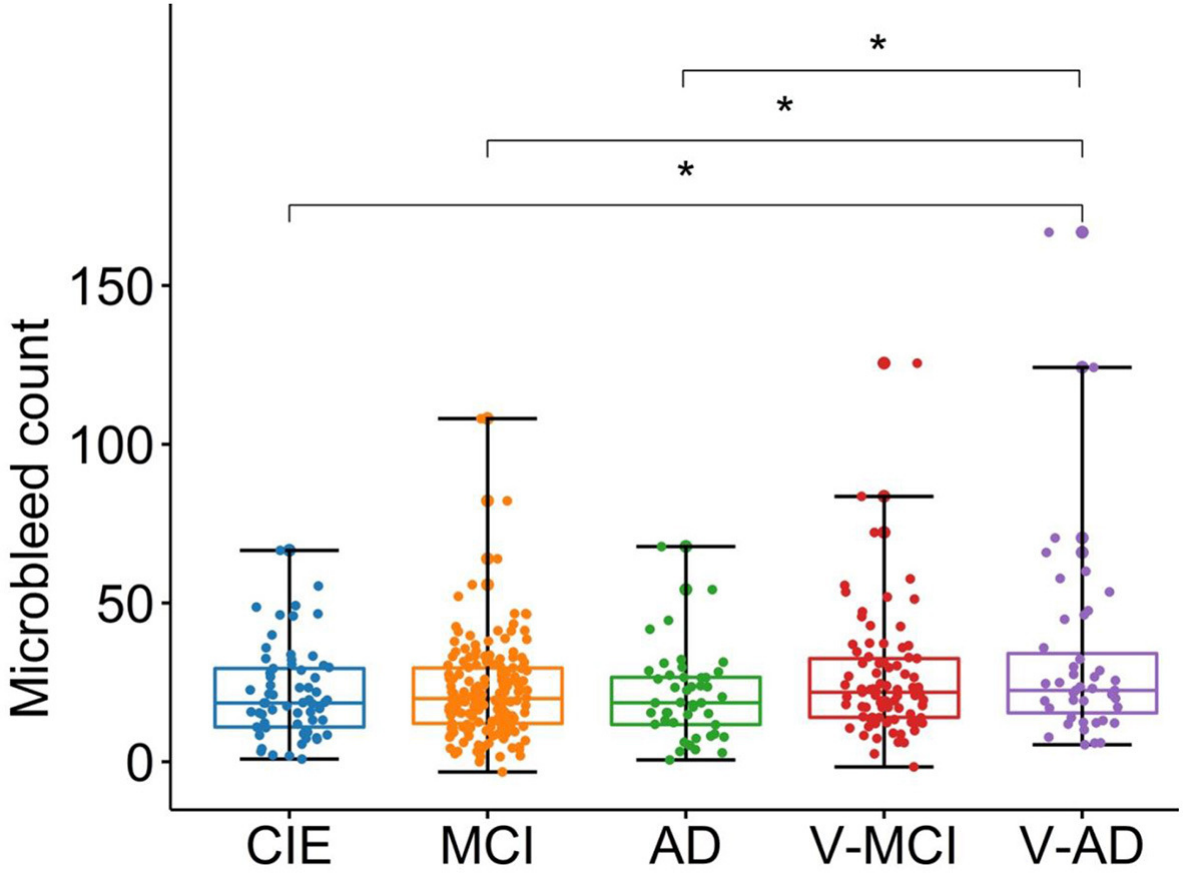
Boxplots showing the adjusted microbleed counts in different cohorts. CIE, cognitively intact elderly; MCI, mild cognitive impairment; AD, Alzheimer's dementia; V-MCI, vascular MCI (V-MCI); (AD), V-AD, vascular AD. Pairwise comparisons were conducted through ANOVA test with the *p*-value adjusted using Bonferroni correction. *P < 0.05, **P < 0.001, ***P < 0.0001. The adjusted microbleed counts were obtained by regressing out the effects of age and sex from the raw microbleed counts, referencing the CIE group.

The adjusted microbleed counts are presented in Table 1 according to diagnostic category. Figure 4 illustrates the corresponding boxplots and pairwise comparisons. There was a significant difference [*F*_(4,400)_ = 4, *P* = 0.005, ANOVA test] between groups in terms of adjusted microbleed counts. Based on the results of ANOVA pairwise comparison, a notable differentiation was observed between CIE vs. V-AD (*P* = 0.028), MCI vs. V-AD (*P* = 0.011), and AD vs. V-AD (*P* = 0.021) with regards to adjusted microbleed counts. Conversely, the remaining pair comparisons did not yield significant outcomes (*P* > 0.05).

### 3.6 Association between brain-PAD and WMH

**Figure 5:**
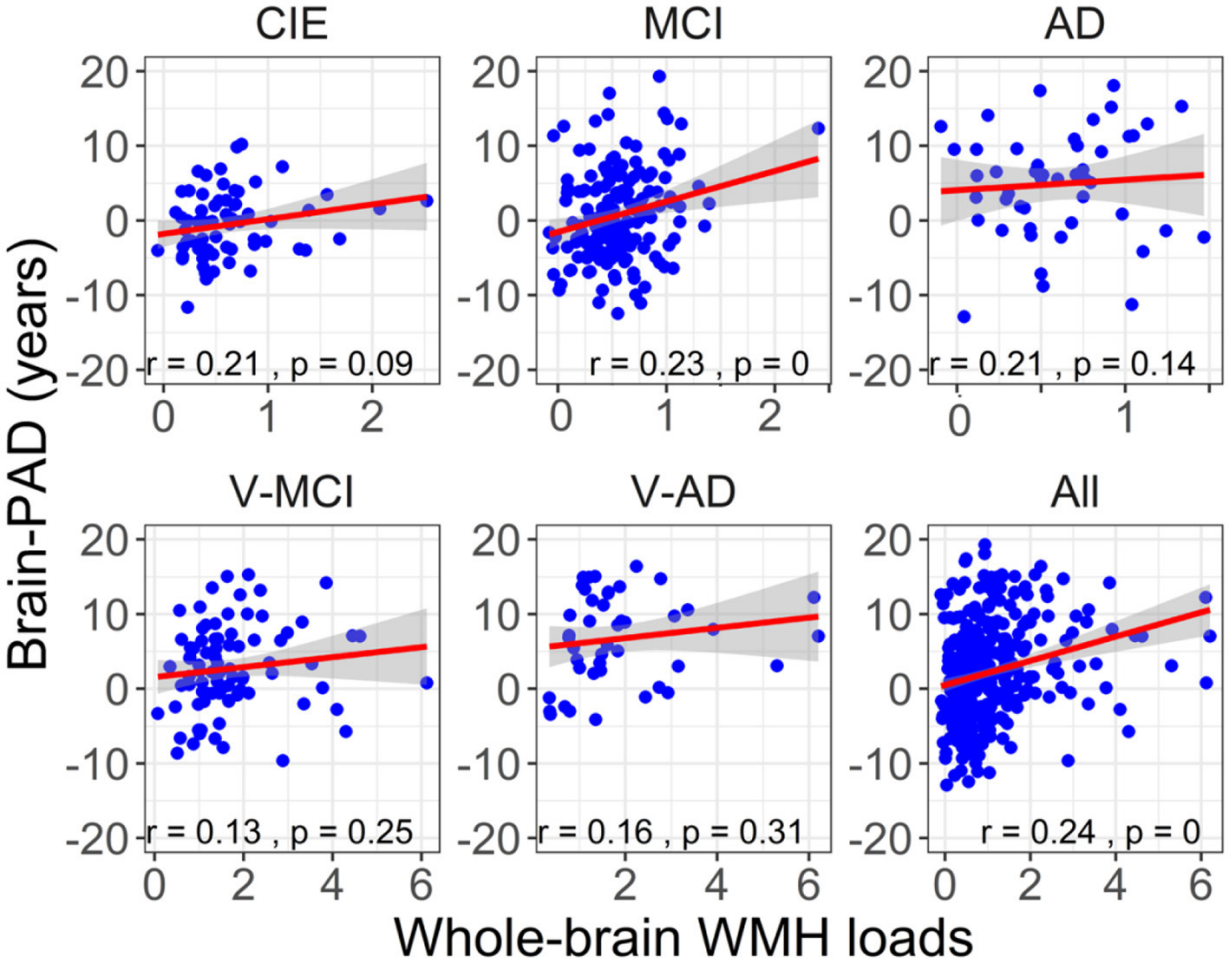
The association between adjusted brain-PAD values and adjusted whole-brain WMH loads in each cohort, as well as in all cohorts. The correlation analysis was conducted using a Pearson correlation test. The brain-PAD values and WMH loads were corrected for age and sex by referencing to the CIE group.

Figure 5 shows the association between brain-PAD and WMH loads in the five categories of participants. Brain-PAD and adjusted WMH loads demonstrated a significant and positive correlation in the MCI cohort as well as all cohorts combined (*r* = 0.24, *P* < 0.001), while in other cohorts this association was found to be marginally insignificant (*P* > 0.05).

### 3.7 Association between brain-PAD and microbleed counts

**Figure 6:**
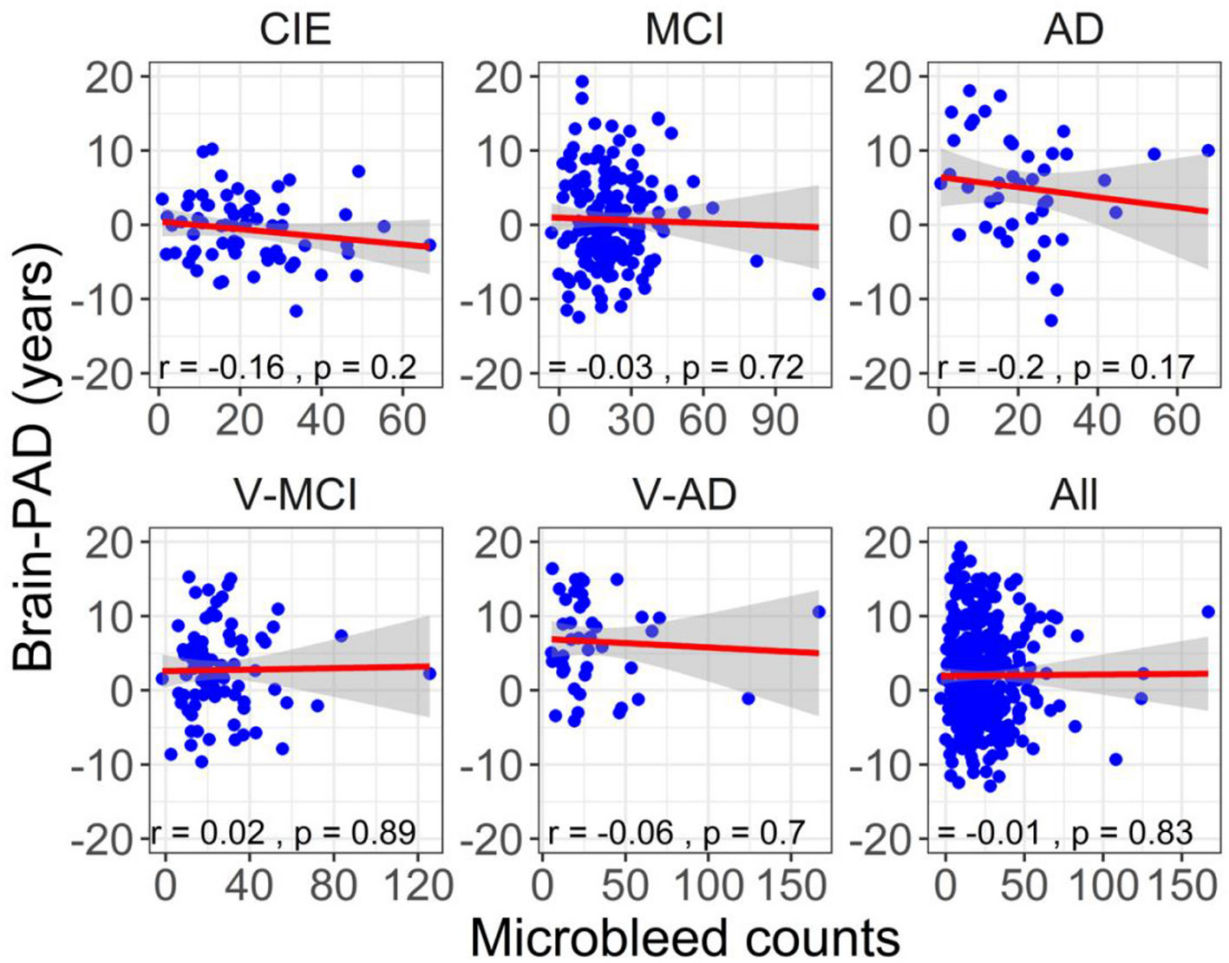
The association between adjusted brain-PAD values and adjusted microbleed counts in each cohort, as well as in all cohorts. The correlation analysis was conducted using a Pearson correlation test. The brain-PAD values and microbleed counts were corrected for age and sex by referencing to the CIE group.

Figure 6 illustrates the correlation between brain-PAD and the number of microbleeds across the five participant groups. There was no statistically significant correlation observed between brain-PAD and microbleed counts in all cohorts.

## 4 Discussion

The primary objectives of this study were 2-fold. First, we sought to assess the impact of cerebrovascular lesion burdens, including WMHs and microbleeds, on brain cortical age and secondly, the relationship between brain-PAD and cerebrovascular lesion loads, both across the aging/cognitive impairment continuum associated with AD. Our results in summary showed that all clinical groups had significantly increased brain-PAD values, except for MCI, with the V-AD group demonstrating the highest mean adjusted brain-PAD of 7.02 ± 6.06 years (Figure 2). Further, our study found a mean adjusted brain-PAD of 5.86 ± 8.02 years in individuals with AD, which aligns with previous research reporting a similar increase of +5 years in brain-PAD among AD patients (Beheshti et al., 2018). However, we observed a lower adjusted mean brain-PAD in our MCI cohort (i.e., 0.70 ± 6.03 years; Table 1) compared to the existing literature (Beheshti et al., 2018). This discrepancy could potentially be explained by the fact that unlike prior studies (Beheshti et al., 2018), we distinguished V-MCI patients from those with MCI, which may suggest more serious MCI cases. To the best of our knowledge, this is the first study which explored brain age among V-MCI and V-AD participants as well, in the same cohort and using a similar imaging protocol.

### 4.1 Impact in cognitively impaired cohorts

Our first hypothesis was that vascular cohorts (V-MCI, V-AD) would experience a significantly accelerated brain aging process compared to non-vascular cohorts (MCI, AD). We observed a significantly higher adjusted brain-PAD in V-MCI than MCI (mean difference = 2.24 years, *P* = 0.04, ANOVA; Figure 2). However, this difference was not statistically significant between AD and V-AD although V-AD showed a higher brain-PAD (mean difference = 1.11 years, *P* > 0.05, ANOVA; Figure 2). These findings suggest that individuals with vascular conditions may experience accelerated brain aging, particularly in the early stages of AD, compared to those without vascular conditions. Further studies of brain age estimation in larger samples are required to confirm this finding.

All participant categories showed a significant WMH load on T2w-FLAIR images (Figure 3). As expected, WMH load in vascular cohorts (i.e., V-MCI, V-AD) were significantly higher than non-vascular cohorts (i.e., CIE, MCI and AD; Figure 3). This finding is in line with other studies which investigated WMH in MCI and AD (Tosto et al., 2014; Desmarais et al., 2021). However, we did not observe a significant difference between V-MCI and V-AD in terms of WMH loads (*P* > 0.05, ANOVA; Figure 3), suggesting that the functional impact from WMH increases due to different processes than simply through an increase in extent, number, or size of lesions.

Our results revealed a statistically significant and positive correlation (*r* = 0.24, *P* < 0.001) between brain-PAD and WMH loads when all groups were combined, suggesting that there is an actual relationship between these two variables rather than one caused by chance. To determine whether the significant correlation between brain-PAD and WMH loads is driven by the MCI cohort (which had a larger sample size), we excluded MCI from the combined groups (*N* = 247). Despite this exclusion, the correlation between brain-PAD and WMH loads remained statistically significant (*r* = 0.19, *P* = 0.002), indicating that this correlation is not solely due to the MCI group. This relationship between WMH and brain-PAD is supported by other evidence that WMH leads to cortical thinning, and therefore may explain changes in cognitive status even though there is a plateau in WMH evolution, as noted above in the V-MCI and V-AD group.

However, when we tested the correlation between brain-PAD and WMH loads within each group, only the MCI cohort showed a significant association between the two variables (*r* = 0.24, *P* < 0.001), whereas the other cohorts did not (*P* > 0.09). The possible explanation is that the sample size within each cohort, except for the MCI cohort, was relatively small compared to the total sample size of all subjects.

We also hypothesized that WMH loads are associated with increasing brain-PAD in the context of AD. With a significant correlation in the MCI cohort (Figure 5), this hypothesis was confirmed only among patients in the early stages of AD. It is noteworthy that, even though a *P*-value of 0.14–0.31 is not statistically significant, it could still be of clinical or practical importance. The lack of significant findings in other AD cohorts can be attributed to the limited number of participants in these groups. Similarly, additional research with larger sample sizes is required to validate our discovery concerning the connection between brain-PAD and WMH in AD across various clinical categories.

### 4.2 Impact in cognitively intact elderly

Based on the literature (Habes et al., 2016), our second hypothesis was that a heavy WMH load can increase brain age in cognitively healthy older adults. We observed a positive relationship between brain-PAD and WMH in the CIE cohort, however, it did not reach a statistically significant level (*r* = 0.21, *P* = 0.09; Figure 5), thus partially negating our initial hypothesis. However, this statistical result is close enough to warrant consideration and might suggest a trend or relationship between the two variables in the CIE cohort, which could be further investigated or validated with more data. A positive association between brain-PAD and WMH in the CIE cohort is in agreement with other studies documenting how a high WMH burden coincides with accelerated brain aging and gray matter atrophy (Habes et al., 2016). Besides, it has been elucidated that the preference of WMH loads among healthy older adults could be associated with impairments in various cognitive domains, such as verbal fluency (Dadar et al., 2022b), learning, memory (Habes et al., 2016), and executive function (Lampe et al., 2019). Taken together, this finding would suggest that WMH should be considered as a potential risk factor for accelerated brain aging among cognitively healthy older adults.

### 4.3 Impact of microbleeds

Consistent with expectations, all categorical cohorts exhibited a notable presence of microbleeds (Table 1), particularly in vascular cohorts (i.e., V-MCI and V-AD). The highest adjusted microbleed count was observed in the V-AD cohort (31.90 ± 30.55), which was significantly higher than that of other cohorts, except for V-MCI (Figure 4). These results highlight the influence of cardiovascular risk factors (e.g., hypertension, total and high-density lipoprotein cholesterol levels, atrial fibrillation, the use of lipid-lowering medications, smoking habits, diabetes, elevated body mass index, and antithrombotic use) on the prevalence of microbleeds in AD patients. Importantly, an elevated number of cerebral microbleeds, particularly in specific brain regions has been shown to correlate with a higher likelihood of experiencing cognitive deterioration and developing dementia (Akoudad et al., 2016). However, insignificant associations between brain-PAD and microbleed counts were detected in all cohorts (Figure 6).

### 4.4 Strengths and limitations

Evidence has shown that younger AD patients tend to exhibit higher levels of brain-PAD compared to older individuals with AD a higher brain-PAD than those who are older (Beheshti et al., 2018, 2021). In our study, we observed differences in the average age among our test groups (Table 1). To account for potential confounding effects from varying age and sex distributions across groups, we conducted regression analyses to remove the influence of age and sex on brain-PAD, WMH, and cerebral microbleed values, using the CIE group (*N* = 70) as the reference point (Equation 1). Given that the CIE group had the youngest age and the highest percentage of female participants (Table 1), adjusting based on this group may introduce bias into our findings and conclusions. To address this concern, we repeated all statistical analyses by controlling for the impact of age and sex on brain-PAD, WMH, and cerebral microbleed values using data from all participants (Supplementary material).

In the test groups, the analysis showed similar significant results for adjusted brain-PAD, except the comparison between the MCI and V-MCI groups, which was not significant (Supplementary Figure S1). For adjusted WMH loads, similar results were found overall (Supplementary Figure S2), but no significant differences were seen between groups regarding adjusted microbleed counts and pairwise comparisons (Supplementary Figure S3). The association between adjusted brain-PAD and the number of microbleeds, as well as between adjusted brain-PAD and WMH loads, showed similar statistical results. However, the association between adjusted brain-PAD and WMH loads in the AD cohorts was significant (Supplementary Figure S4). The fact that the links between WMH and brain-PAD persist after correcting for age and sex suggests that the relationship between these two features is not solely determined by variations in age and sex across groups. There are certainly a few confounders that could explain these associations that were not explored in this study (e.g., cardiovascular risk factors, genetics, amyloid and tau positivity). However, this may also be a consequence of potential coupled temporal dynamics between WMH and gray matter degeneration (Dadar et al., 2022a; Garnier-Crussard et al., 2023). While we accounted for the impact of age and sex in our statistical analysis, it remains vital to acknowledge and address this point to prevent biases in interpreting the results, especially when comparing different clinical groups.

We highlight the fact that our brain age prediction model was created using data from multiple sites and scanners (Section 2.2), and then applied to the independent test datasets. This fact demonstrates the generalizability of our findings, indicating the capacity of our predictive model to perform on new data from the same population, even if it was not included in the original training phase. In MRI pre-processing stage, we employed a validated software, specifically *FreeSurfer*, renowned for its suitability in multi-center and multi-scanner studies (Dadar et al., 2020). Moreover, in order to reduce any potential impact of various scanner manufacturers on *FreeSurfer* measurements, we incorporated a validated harmonization technique known as ComBat (Fortin et al., 2018; Torbati et al., 2021). To ensure the separation of brain features extracted by *FreeSurfer* from WMH (Dadar et al., 2021b), only cortical brain features were used in our brain age prediction model.

## 5 Conclusion

This study aimed to explore how cerebrovascular lesion loads affect brain health in in the context of aging and various forms of AD, as well as the connection between brain-PAD and cerebrovascular lesion loads. Our findings indicated that the presence of cerebrovascular lesion loads could hasten brain aging in AD patients. Additionally, our results demonstrated a possible link between brain-PAD and WMH loads, indicating a strong association between WMHs and accelerated brain aging, resulting in an older-appearing brain. Although some of the clinical cohorts used in this study did not show statistically significant associations, the clinical relevance of the observed trend could be noteworthy. Conversely, there was no significant correlation observed between brain-PAD and microbleed loads. Taken together, it can be inferred that the presence of WMH loads has the potential to significantly accelerate brain aging not only in the context of AD but also among cognitively healthy older adults, while the impact of microbleed loads may not be as significant.

In spite of the fact that these links may differ based on the diagnosis, these findings indicate the importance of treatment and prevention strategies for vascular risk factors (e.g., lifestyle changes, anti-hypertensive medications, lipid-lowering treatments, blood sugar management, and exercise), which might be able to slow down the progression of cerebrovascular lesions and delay the effect on cortical thickness. Future research studies may aim to assess the efficiency of different WMH treatments and prevention strategies in the area of brain aging.

## Data availability statement

The original contributions presented in the study are included in the article/Supplementary material, further inquiries can be directed to the corresponding author.

## Ethics statement

Each database was approved by an Ethics Committee for human experimentation before study commenced, and the participants provided written informed consent for the studies involving humans because data used in this article were obtained from various datasets, including the Comprehensive Assessment of Neurodegeneration and Dementia (COMPASS-ND) cohort of the Canadian Consortium for Neurodegeneration and Aging (CCNA), Alzheimer's Disease Neuroimaging Initiative (ADNI), Alzheimer's Disease Repository Without Borders (ARWIBO), and The Open Access Series of Imaging Studies (OASIS). The studies were conducted in accordance with the local legislation and institutional requirements. The participants provided their written informed consent to participate in this study.

## Author contributions

IB: Conceptualization, Data curation, Formal analysis, Investigation, Methodology, Software, Validation, Visualization, Writing – original draft, Writing – review & editing. OP: Conceptualization, Data curation, Methodology, Resources, Writing – review & editing. MD: Data curation, Methodology, Resources, Writing – review & editing. SD: Conceptualization, Formal analysis, Funding acquisition, Project administration, Resources, Supervision, Writing – review & editing.
