# Supplementary material for: Cerebrovascular lesion loads and accelerated brain aging: insights into the cognitive spectrum
Source: Front Dement. 2024 Jun 21;3:1380015. doi: 10.3389/frdem.2024.1380015 (PMC11285662; doi:10.3389/frdem.2024.1380015)
Supplement: Supplementary file 1 [file Table_1.DOCX]

Supplementary Information

**In this section, we report the key results, accounting for the impact of age and sex on brain-PAD, WMH, and cerebral microbleed values, using data from all participants.**

**S.1 Demographics**

Table S1. Clinical demographics, WMH load, Microbleed count, and brain-PAD by diagnosis by controlling for the impact of age and sex using data from all participants.

|  | CIE  (N=70) | MCI  (N=173) | AD  (N=50) | V-MCI  (N=88) | V-AD  (N=47) | *P* |
| --- | --- | --- | --- | --- | --- | --- |
| Female (%) | 78% | 45% | 36% | 34% | 49% | < 0.0001 |
| Real age (yrs) | 69.8 ± 6.6 | 71.8 ± 6.6 | 73.9 ± 8.2 | 76.1 ± 5.5 | 76.7 ± 6.3 | < 0.0001 |
| MoCa | 27.7±1.55 | 23.61±3.12 | 18.68±3.72 | 23.15±3.22 | 17.78±3.30 | < 0.0001 |
| WMH load^†^ | 0.61±0.5^a^ | 0.63±0.36 ^c^ | 0.79±0.37 | 1.97±1.16 ^f^ | 2.16±1.38 ^f^ | < 0.0001 |
| WMH load^*^ | 0.90±0.48^a^ | 0.70±0.43 ^c^ | 0.71±0.53 | 1.77±1.04 ^f^ | 1.93±1.78 ^f^ | < 0.0001 |
| Microbleed count^†^ | 21.52±14.24 ^b^ | 23.78±14.06 ^d^ | 22.06±13.14 ^e^ | 27.12±18.96 ^f^ | 32.67±31.50 ^e^ | 0.01 |
| Microbleed count^*^ | 25.14±14.32 ^b^ | 23.85±14.92 ^d^ | 21.12±11.79 ^e^ | 25.44±18.65 ^f^ | 31.20±30.63 ^e^ | 0.078 |
| Brain-PAD  (yrs) ^†^ | -0.52 ±4.75 | 1.89 ± 5.5 | 7.22±7.33 | 3.97±5.43 | 7.64±5.95 | < 0.0001 |
| Brain-PAD  (yrs) ^*^ | -0.19±4.73 | 1.95±5.58 | 7.16±7.48 | 3.77±5.40 | 7.44±6.08 | < 0.0001 |

CIE: Cognitively Intact Elderly, AD: Alzheimer's Disease, MoCA: Montreal Cognitive Assessment, MCI: Mild Cognitive Impairment, V-AD: Vascular Alzheimer's Disease. All variables are presented based on the mean ± standard deviation. *P*: The results of an ANOVA for continuous variables and a Chi-square test for categorical variables between groups. ^†^ Raw data;

^*^Sex- and age-adjusted data by referencing all participants.

^a^Data missing in two participants.

^b^Data missing in five participants.

^c^Data missing in thirteen participants.

^d^Data missing in seven participants.

^e^Data missing in four participants.

^f^Data missing in three participants.

**S.2 Brain age estimation on the test set**

The mean brain-PAD values are shown in Table S1 and Figure S1. There was a significant difference in adjusted brain-PAD values [F (4,423) = 20, *P* < 0.001, ANOVA test] among groups. All categories of patients exhibited a significantly higher mean adjusted brain-PAD than the CIE group (*P* < 0.001), except for the MCI cohort (*P* = 0.085). The V-AD cohort had the highest adjusted brain-PAD. Post hoc pairwise group comparison based on the ANOVA test showed statistically significant differences (*P* < 0.05) in terms of adjusted brain-PAD between pair groups, except for CIE vs. MCI, MCI vs. V-MCI, and V-AD vs. AD (*P* > 0.05).

| 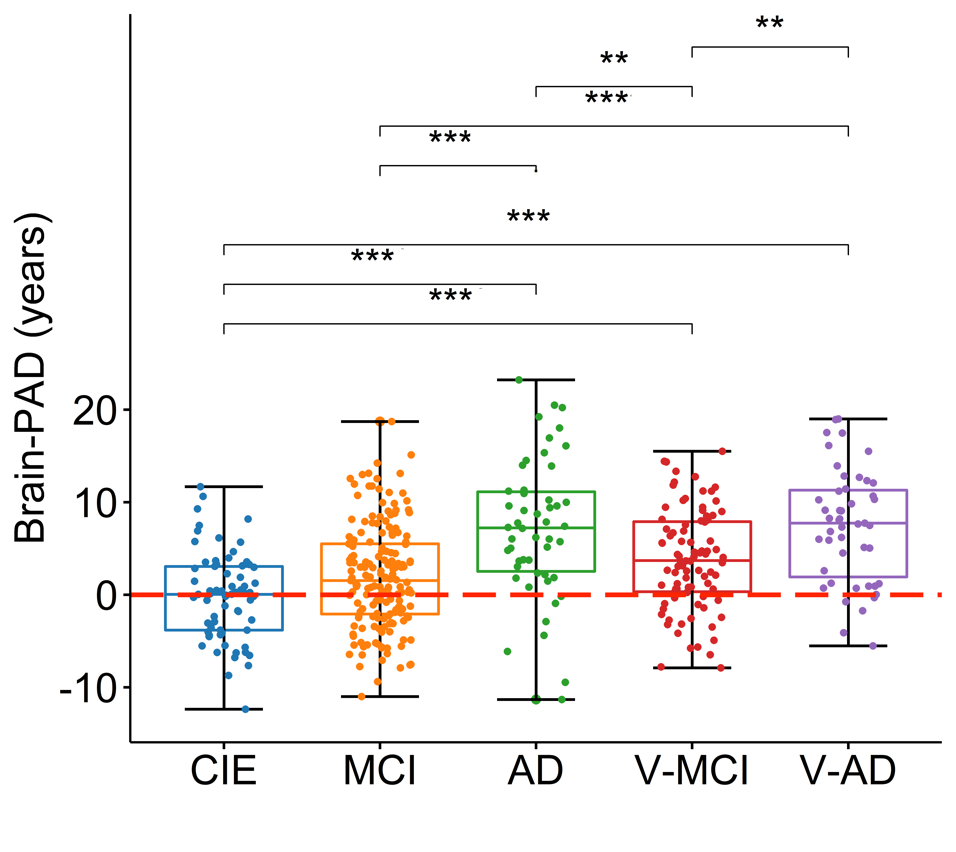 |
| --- |
| **Figure S1**: Boxplots depicting the adjusted brain-PAD values among different cohorts. CIE: cognitively intact elderly, MCI: mild cognitive impairment, AD: Alzheimer’s dementia, V-MCI: vascular MCI (V-MCI), (AD), V-AD: vascular AD. Pairwise comparisons were conducted through ANOVA test with the p-value adjusted using Bonferroni correction. * *P* < 0.05, ** *P* < 0.001, *** *P* < 0.0001. Note: The adjusted brain-PAD values were obtained by regressing out the effects of age and sex from the raw brain-PAD values, referencing all participants. |

**S.3 WMH loads**

Table S1 summarizes adjusted WMH loads by diagnostic category, whereas Fig. S2 shows respective boxplots as well as pairwise comparisons. As could be expected, there was a significant difference in WMH loads [ F (4,402) = 44, *P* < 0.001, ANOVA test] between groups. Unsurprisingly, both V-MCI and V-AD showed a significantly higher WMH load compared to non-vascular groups (i.e., CIE, MCI and AD) in terms of adjusted WMH loads by ANOVA pairwise comparison (*P* < 0.001). However, there were no pairwise differences between CIE vs. MCI, CIE vs. AD, MCI vs. AD, and V-MCI vs. V-AD (*P* > 0.05).

| 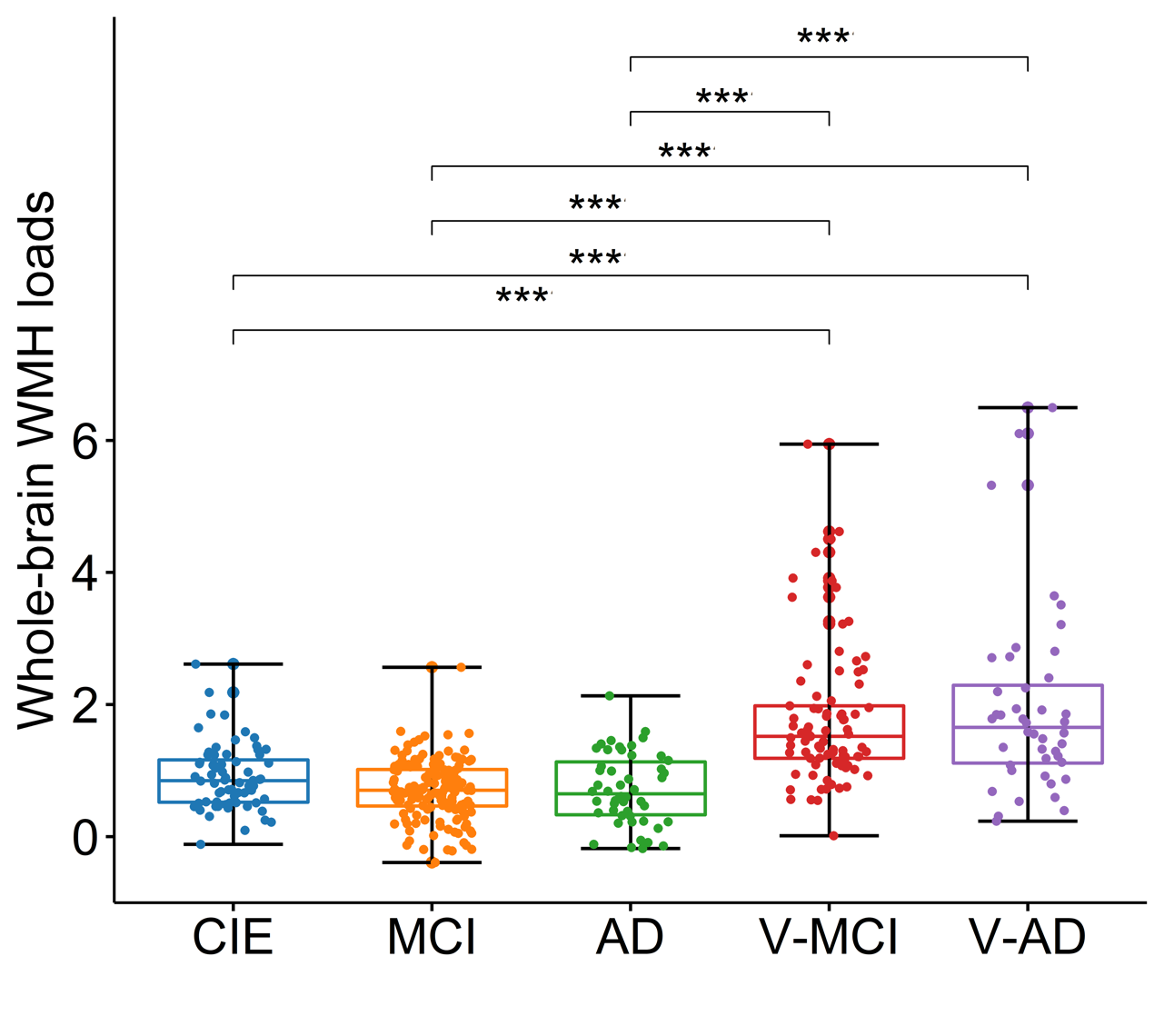 |
| --- |
| **Figure S2:** Boxplots depicting the adjusted WMH loads among different cohorts. CIE: cognitively intact elderly, MCI: mild cognitive impairment, AD: Alzheimer’s dementia, V-MCI: vascular MCI (V-MCI), (AD), V-AD: vascular AD. Pairwise comparisons were conducted through ANOVA test with the p-value adjusted using Bonferroni correction. * *P* < 0.05, ** *P* < 0.001, *** *P* < 0.0001. Note: The adjusted WMH loads were obtained by regressing out the effects of age and sex from the raw WMH loads, referencing all participants. |

**S.4 Microbleed counts**

The adjusted microbleed counts are presented in Table S1 according to diagnostic category. Figure S3 illustrates the corresponding boxplots and pairwise comparisons. There was a significant difference [ F (4,400) = 2, *P* = 0.078, ANOVA test] between groups in terms of adjusted microbleed counts. Based on the results of the ANOVA pairwise comparison, the pair comparisons did not yield significant outcomes (*P* > 0.05).

| 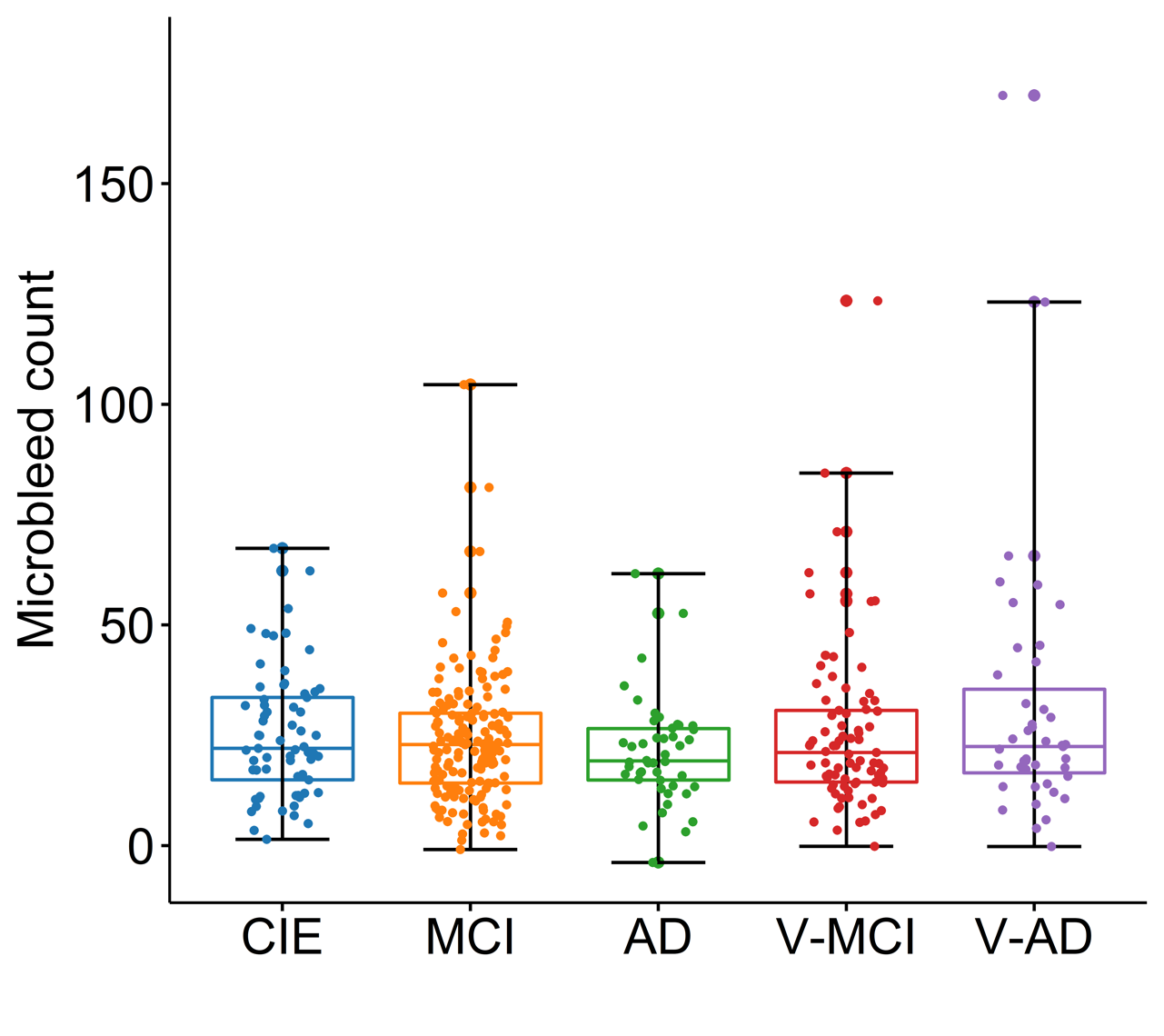 |
| --- |
| **Figure S3:** Boxplots showing the adjusted microbleed counts in different cohorts. CIE: cognitively intact elderly, MCI: mild cognitive impairment, AD: Alzheimer’s dementia, V-MCI: vascular MCI (V-MCI), (AD), V-AD: vascular AD. Pairwise comparisons were conducted through ANOVA test with the p-value adjusted using Bonferroni correction. * *P* < 0.05, ** *P* < 0.001, *** *P* < 0.0001. Note: The adjusted microbleed counts were obtained by regressing out the effects of age and sex from the raw microbleed counts, referencing the all participants. |

**S.5 Association between brain-PAD and WMH**

Figure S4 shows the association between adjusted brain-PAD and WMH loads in the five categories of participants. Brain-PAD and adjusted WMH loads demonstrated a significant and positive correlation in the MCI and AD cohorts as well as all cohorts combined, while in other cohorts this association was found to be marginally insignificant (*P* > 0.05).

| 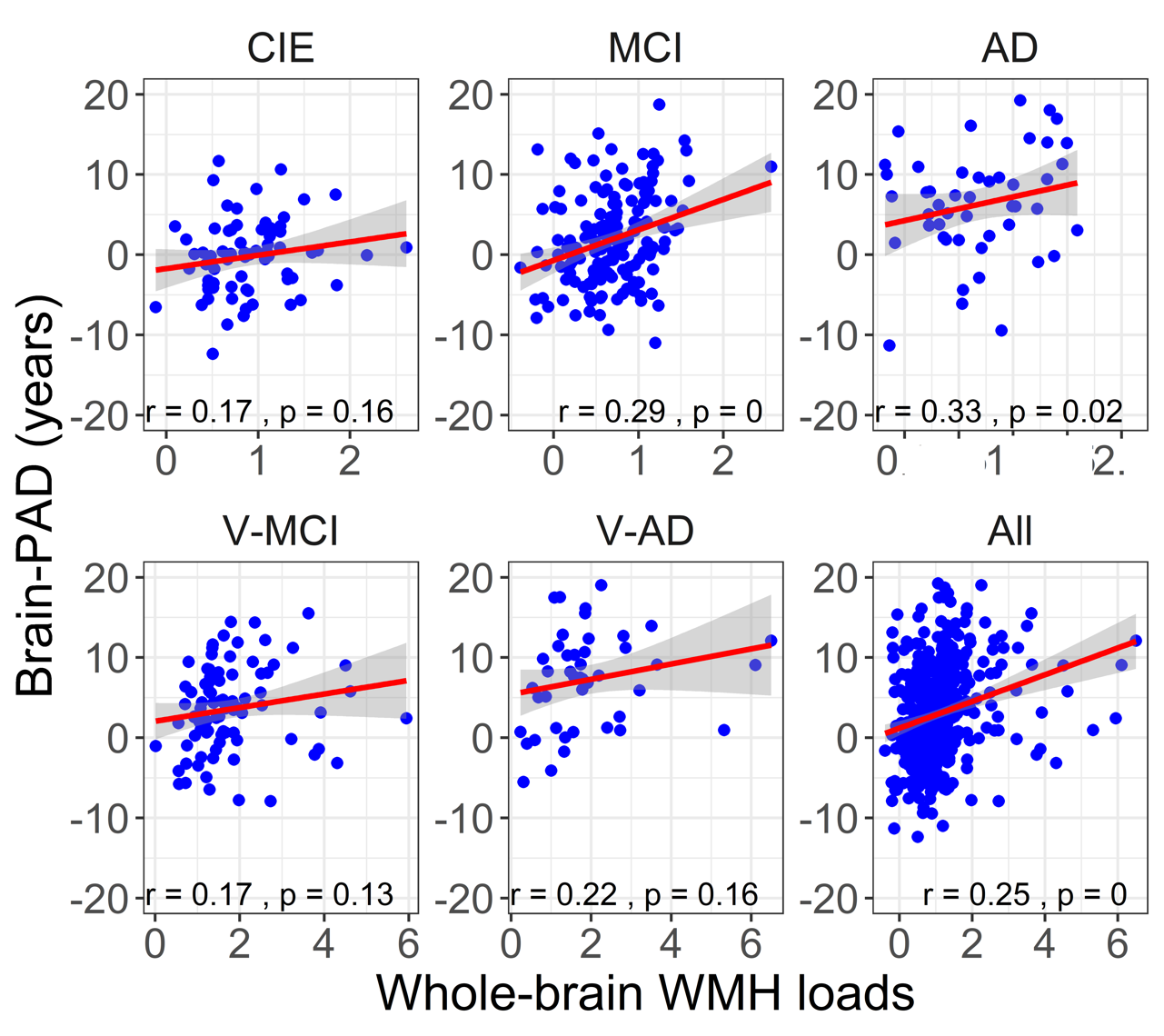 |
| --- |
| **Figure S4:** The association between adjusted brain-PAD values and adjusted whole-brain WMH loads in each cohort, as well as in all cohorts. The correlation analysis was conducted using a Pearson correlation test. The brain-PAD values and WMH loads were corrected for age and sex by referencing to all participants. |

**S.6 Association between brain-PAD and microbleed counts**

Figure S5 illustrates the correlation between adjusted brain-PAD and the number of microbleeds across the five participant groups. There was no statistically significant correlation observed between brain-PAD and microbleed counts in all cohorts.

| 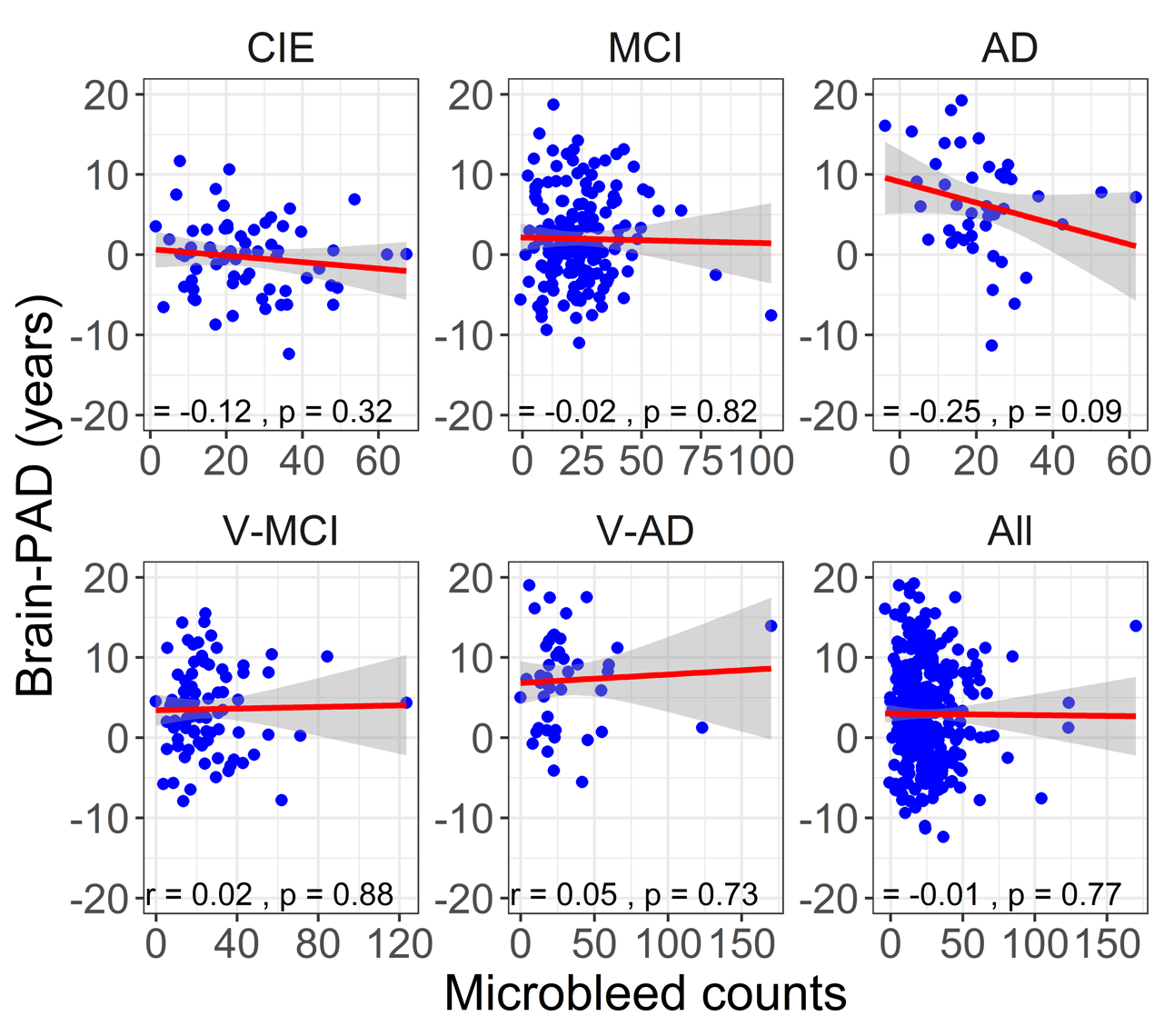 |
| --- |
| **Figure S5:** The association between adjusted brain-PAD values and adjusted microbleed counts in each cohort, as well as in all cohorts. The correlation analysis was conducted using a Pearson correlation test. The brain-PAD values and microbleed counts were corrected for age and sex by referencing all participants. |
